# Mobile Apps for the Management of Comorbid Overweight/Obesity and Depression/Anxiety: A Systematic Review

**DOI:** 10.1155/2020/9317179

**Published:** 2020-01-23

**Authors:** Lizzette Gómez-de-Regil, Azalia Avila-Nava, Ana Ligia Gutierrez-Solis, Roberto Lugo

**Affiliations:** Hospital Regional de Alta Especialidad de la Península de Yucatán, Calle 7, No. 433 por 20 y 22, Fraccionamiento Altabrisa, Mérida 97130, Yucatán, Mexico

## Abstract

**Objective:**

This review aimed at searching for scientific literature on mobile apps for the management of comorbid overweight/obesity and depression/anxiety and providing a brief and comprehensive summary of their main features, targeted groups, and relevant results.

**Methods:**

A bibliographical search was performed in Pubmed, PsycNet, Web of Science, ResearchGate, and Lilacs databases. The terms “obesity” and “overweight” were introduced in combination with “anxiety” and “depression” and “mobile app (application),” “smartphone app (application),” “android app (applicattion),” “iOS app (application),” “mobile health app (application),” and “mHealth app (application).”

**Results:**

The initial search eliciting 204 citations was reduced to 7 relevant papers (4 original articles, 1 brief communication, and 2 study protocols). All publications were from the last five years, most were produced by research teams from the United States. All had adult samples, and interventions mostly followed a cognitive behavioral framework. Regarding mobile apps, five studies only used one to monitor weight and physical activity, one study to provide therapy to improve psychological wellness, and one study to monitor cognitions and emotions. No mobile app was found for the simultaneous management of overweight/obesity and depression/anxiety.

**Conclusions:**

The prevalence and costs related to overweight/obesity and depression/anxiety are significant and likely to increase. Very often these conditions overlap; thus, it would be recommendable to treat their comorbidity simultaneously. Nevertheless, no mobile app has been designed for this purpose, which would help to reduce service provision costs and make treatment more easily accessible for patients.

## 1. Introduction

During the last decades, technology has driven societies into an accelerated process of change. Users are provided with easy and virtually infinite access to information. Learning and working have gradually become more dependent on the use of electronic tools and computer programs. This has made human tasks more efficient and effective while also promoting a rather unhealthy life style. New technologies and electronic-based activities have produced a decrease in physical activity, more sedentary behavior, and poorer dietary patterns, all acknowledged as major behavioral determinants of obesity [1]. Basically, obesity and overweight are the outcome of an energy imbalance between calories consumed and calories expended [2], and over the last decades, their prevalence has doubled and even quadrupled [3]. These pathologies increase the risk of chronic diseases, mainly type 2 diabetes, dyslipidemia, hypertension, cardiovascular diseases, cancer, and musculoskeletal disorders, these conditions being responsible for the vast majority of deceases worldwide [2, 4, 5].

Moreover, excess body weight is also known to be associated with increased prevalence of mental disorders [6–9]. This association seems influenced by gender, age [7, 9–11], and even race/ethnicity [8, 11, 12]. For instance, McCrea and colleagues [7] found that, in young women, having a disorder increased along with Body Mass Index (BMI), whereas in young men, probabilities were higher for both underweight and obese men; but these associations diminished in older age groups. Among various mental disorders, depression and anxiety stand out, not only for their higher prevalence but also for their marked association with overweight/obesity [13–15]. Although a bidirectional link has been repeatedly observed [11, 16–19], there are also some discrepancies in findings that might be attributed to the effects of gender [20], the criteria to measure obesity [21], and the selected mental disorder instruments [14]. The link rather than linear seems to follow a U-shaped pattern, with stronger associations in the underweight and obesity groups [7, 12, 22]. Evidence, mainly from cross-sectional studies, supports a significant association between anxiety/depression and increased overweight/obesity, although no causal relationship can be inferred in either direction [19, 23]. To the best of our knowledge, public survey data of the prevalence of mental health disorders and overweight/obesity coexistance are not available; yet, systematic reviews have provided some estimates regarding the strength of relationship between obesity and psychiatric disorders. Luppino and colleagues [11] concluded that obese people had a 55% increased risk of developing depression over time, whereas people with depression had a 58% increased risk of becoming obese. Rajan and Menon [20] estimated the odds ratios (ORs) to be similar for developing depression in obesity (OR: 1.21–5.8) and vice versa (OR: 1.18–3.76) with a stronger association observed in women. For anxiety disorders, they found ORs to be less strong (OR: 1.27–1.40). Gariepy and colleagues [19] estimated a pooled OR of an association between obesity and anxiety of 1.40.

On the other hand, technology can also support the achievement of health objectives. Mobile health (mHealth), defined by the Global Observatory for eHealth as the “medical and public health practice supported by mobile devices, such as mobile phones, patient monitoring devices, personal digital assistants (PDAs), and other wireless devices” [24], has the potential to influence on a variety of health outcomes and has become a key trend in health service provision during the last years [25]. Psychosocial and health behavior interventions can now reach a bigger number of patients and from more distance locations by using mobile technology during their everyday lives (i.e., in real time) and in natural settings (i.e., real world) [26]. Mobile applications, known as “apps,” facilitate remote access to health services, connecting patients with health professionals around the world in a safe, private, and confident manner, offering results in short terms [27, 28].

A meta-analysis performed by Qudah and Luetsch [28] found that more than 325 thousand health-related apps for iOS and Android platforms are available. Among the most targeted health conditions were obesity and dietetics, mental health disorders, diabetes, asthma, cancer, chronic kidney disease, chronic obstructive pulmonary disease, Parkinson's disease, pregnancy-prenatal care, and rehabilitation. The number of apps for weight loss/management has increased rapidly. However, most commercial mobile apps for weight loss/management seem suboptimal in quality: inadequate scientific coverage, inaccurate weight-related information, lack important evidence-based features, do not involve health-care experts in their development process, overlook behavior change techniques, and have not undergone rigorous scientific testing [29–32]. Numerous mobile apps aiming at managing depression and anxiety symptoms are also available. Although most of them have not been rigorously designed and tested, there is some promising evidence of their effectiveness that needs to be validated [33–35].

The epidemic of overweight and obesity is expanding rapidly and to younger ages; thus, it has become a major challenge to healthcare systems due to their high economic and psychosocial burden [3, 4, 36, 37]. According to the 2017 Obesity Update released by the Organization for Economic Cooperation and Development (OECD), Mexico has an obesity rate of 33.3%, only surpassed by the United States and Chile, and it is projected to rise to 39% in 2030. Considering overweight and obesity in people aged 15–74 years, Mexico has the highest rate, 72.5% [38]. The most recent national health survey reported overweight/obesity prevalence of 71.2% in adults, 36.3% in adolescents, and 33.2% in school-aged children [39].

On the other hand, depression and anxiety are the most common mental disorders, with prevalence rates (in 2015) of 4.4% and 3.6% on global population, respectively [40]. In Mexico, anxiety disorders, followed by mood disorders (including depression), have been reported with the highest prevalence in adult populations, with rates of 14.3% and 9.2%, respectively [41]. It has been estimated that depression accounts for a global total of over 50 million Years Lived with Disability (YLD), and anxiety disorders led to a global total of 24.6 million YLD [40]. Direct and indirect costs of depression and anxiety make these disorders main contributors to the global burden of disease [42, 43].

Given the high prevalence of these disorders reported in Mexico, overweight/obesity and depression/anxiety are public health priorities demanding cost-effective interventions to reduce their burden. Given the extent evidence supporting their association, it might be worth considering the provision of a combined mHealth treatment for both conditions through the use of a mobile app. This review aimed at searching for scientific literature on mobile apps for the management of comorbid overweight/obesity and depression/anxiety and providing a brief and comprehensive summary of their main features, targeted groups, and, if available, relevant results. The focus was on finding available mobile apps built upon scientific foundations, regardless of their features, empirical evidence, target population, or interventional level (i.e., prevention or alleviation of comorbidity). Findings would point the way for the design, development, and testing of an intervention program aiming at attending simultaneously overweight/obesity and depression/anxiety through the use of mobile apps.

## 2. Method

For the first time, a bibliographical search was performed on the topic consulting the Pubmed, PsycNet, Web of Science, ResearchGate, and Lilacs databases. The terms “obesity” and “overweight” were introduced in combination with “anxiety” and “depression” and “mobile app (application),” “smartphone app (application),” “android app (applicattion),” “iOS app (application),” “mobile health app (application),” and “mHealth app (application).” Inclusion criteria were (1) research papers, (2) published in peer-reviewed journals, (3) available in English or Spanish, and (4) published during the last decade (2009–2019). Exclusion criteria were (1) reviews and/or meta-analyses, (2) papers exclusively discussing theoretical perspectives, (3) empirical studies not reporting on mobile apps, and (4) empirical studies not reporting on the use of mobile apps for the purpose of overweight/obesity and depression/anxiety management. Papers reporting study protocols were agreed to be included, given that, although they do not provide empirical evidence of the use of the mobile apps, they may offer a detailed description of the development of the application and the study design. Online consultations proceeded from the 13th to the 17th of May, 2019.

Citations to works other than research articles (i.e., responses, conference reports, and books) were first withdrawn. Following this, original research was filtered and (systematic) reviews and/or meta-analysis were omitted, as well as theory-based papers. Through the information provided in the abstracts of the remaining material, research not focusing on the use of mobile apps was excluded. According to the study objective, the relevance of each publication was verified and only publications reporting on the use of mobile apps for the management of comorbid overweight/obesity and depression/anxiety were selected. Finally, the following features were recorded from each article: authors, year of publication, location of research team, type of manuscript, name of the project (if available), design, number of groups, objectives, sampling criteria, number and main features of participants, intervention, use of mobile app, outcome measures, and main results. All four authors worked together through the procedure; discrepancies were minimal.

## 3. Results

**Figure 1:**
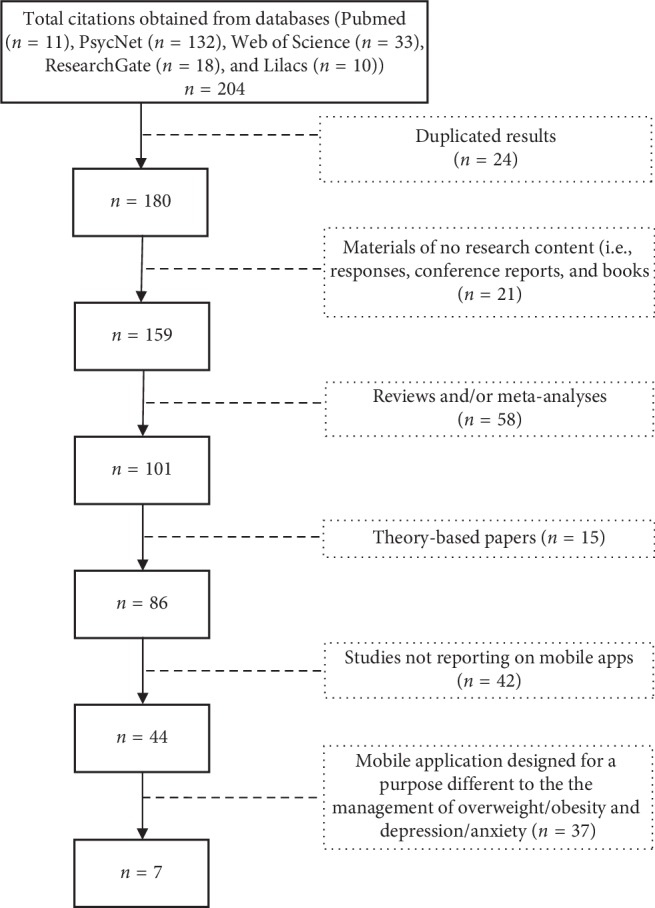
Study flow diagram.

The initial search from the five selected databases elicited 204 citations with 24 duplicated. Through the review of available abstracts, the list reduced to 7 relevant papers (Figure 1).

Table 1 presents the manuscripts' basic identification and design features. All were published from 2015 to 2019, and most were original articles. All but one publication (from Finland) is from four different research groups in the United States.

Table 2 summarizes the publications' main features. All studies included adult patients with overweight or obesity. One study focused on pregnant women with no history of psychosis or depression. Another study included people with no clinical diagnosis of mental disorder but with psychological distress. The studies by Williams et al. [44], Ma et al. [45], and their colleagues included patients with clinically significant depression, the studies by Naslund and colleagues [46, 47] included patients with diagnoses of severe mental health illness (i.e., schizophrenia, bipolar disorder, major depressive disorder), and the study by Levinson and colleagues [48] included exclusively patients with eating disorders. Actual sample sizes ranged from 10 (pilot study with patients with severe mental illness recruited from one community mental health centre) to 219 (randomized control trial including people with psychological distress recruited by advertisements in local newspapers from three Finnish cities). The study protocols published by the RAINBOW/ENGAGE group aimed at recruiting 100 to 404 participants. Mean age of participants ranged from 24.9 to 50.2.

Excluding the descriptive study, all six designs relied on behavioural management interventions intended for the target samples. For instance, the SmartMoms intervention for pregnant women, the Acceptance and Commitment Therapy (ACT) for the psychologically distressed, the I-CARE program for comorbid obesity and depression, and the In SHAPE for weight loss adapted to people with severe mental illness.

Regarding the use of mobile apps, the studies by Ma et al. [45], Naslund et al. [46, 47], and their colleagues included apps to monitor physical activity alone or with dietary intake: MyFitnessPal, Fitbit, and Nike FuelBan; the last two were designed to synchronize with their corresponding physical devices. The study by Levinson and colleagues [48] described a mobile app aiming at keeping track of not only comportments but also cognitions and emotions in relation to eating disorders, behaviours, and anxiety. The ENGAGE study would use the Mindstrong app to collect data on participants' use of smartphones (e.g., patterns of keyboard typing, screen swiping and tapping, search terms, and interaction by phone calls); these data will be, through algorithms, converted to specific psychometric tasks capable of assessing neuropsychological capacities. The Expecting Success study did not provide much information regarding the mobile app, and its purpose seems limited to providing remote behavioural weight management counselling through a smartphone, as counterpart to a face-to-face interaction.

Considering those studies where the individuals' outputs were the focus, internal outcomes relate to various psychosocial variables including quality of life, perceived stress, functioning/disability, and symptoms of depression, anxiety and eating disorders, whereas external outcomes were mainly weight, BMI, and physical activity. One study assessed the feasibility and acceptability of a mobile app among individuals with serious mental illness, while other proposed data collection to develop algorithms for neuropsychological tasks.

Regarding the studies' main outcomes, Naslund and colleagues [47] found patients with severe mental illnesses to be highly satisfied and motivated with the use of a mobile app to improve their physical activity and even their social interactions. Levinson and colleagues [48] identified some cognitions (e.g., worry about gaining weight) that could predict higher anxiety before, during, and after the meal and eating disorder pathology. Regarding interventional designs, Järvelä-Reijonen and colleagues [49] reported benefits of Acceptance and Commitment therapy on eating behaviours in people with psychological distress; for instance, increment in eating for physical rather than emotional reasons and decrement in uncontrolled eating and using food as a reward. Also, Naslund and colleagues [47] reported that their lifestyle behavioural intervention on patients with severe mental illness increased physical activity (i.e., step count) and weight loss, although not fitness. Altazan and colleagues [50] found in the group of pregnant women an association between higher weight gain and worse mood and an increment of depressive symptoms over time; yet, regarding the SmartMoms intervention, no significant effects were found on mood and quality of life.

Although not initially considered, it is worth to mention some features regarding the quality of the designs. The two studies by Naslund and colleagues [46, 47] and the one by Levinson and colleagues [48] were neither randomized nor blinded. Although the other four studies [44, 45, 49, 50] were randomized controlled trials, only the study by Ma and colleagues [45] was blind. Although the target populations of the studies were diverse (e.g., pregnant women and people with an eating disorder or a severe mental illness), according to their reports, a common bias is the recruitment by convenience sampling, which limits the generalization of their results. Retention rates were mentioned in all but one [48] empirical study, ranging from 79.1% [47] concluding a 6-month intervention to 93.6% and 97.6% assessed at 10 and 36 weeks after the baseline and having concluded a 8-week intervention [49].

## 4. Discussion

Overweight/obesity and depression/anxiety are physical and mental public health priorities due to their high and increasing prevalence and their substantial direct and indirect costs. Research has repeatedly found a strong association between increased BMI and mental health problems [6, 8, 9, 13], though the direction of influence is still unclear [11, 51]. People with depression or anxiety might present significant weight gain due to irregular eating patterns and sedentary lifestyles triggered by clinical symptoms and/or the medications to treat them. A tendency to overeat in response to negative emotions, known as emotional eating, is often observed in depression and anxiety and highly associated to weight outcomes, both in respect to weight gain over time and difficulties with weight loss and weight loss maintenance [52]. Thus, weight management on emotional eaters should not focus on calorie-restricted diets but rather on emotion regulation skills [53].

From the last decades the use of smartphones and mobile apps has exponentially expanded. Along, mHealth services have spread making it possible to provide attention in a remote way, to a larger number of patients and at a lower cost. Numerous mobile apps are available on the market to help users with the management of health issues, including weight loss and mental health difficulties [26, 54]; yet, most lack scientific foundation and evidence. For people with overweight/obesity and depression/anxiety and important health issues that are strongly associated, it would be advisable to use a mobile app designed for their simultaneous management. This study aimed at searching for scientific literature on this topic. Publications are still scarce, produced during the last five years and mainly from the United States. No research on the topic has been published from Mexico regardless of the significant prevalence of these conditions. This epidemiology calls for research and clinical efforts to provide efficient services targeting comorbidities, and the use of mobile technology might well serve to this purpose, particularly when working with young populations more familiarized with mobile apps.

Interventions mostly followed a cognitive behavioral framework. This type of therapy focuses on identifying and modifying unhelpful cognitions and behaviors to improve emotional regulation and produce more convenient solutions to problems. The selected publications provide evidence of its feasibility and efficacy to manage weight and mental disturbances through mobile apps, although to the best of our knowledge, no mobile app has been developed and tested as a simultaneous intervention for both aspects. Relying on the vast literature on cognitive behavioral therapy, it would be recommended to design a mobile app intervention for managing comorbidity, considering the targeted age group (i.e., adults, adolescents, or children) and the presence of psychiatric symptoms on a clinical or subclinical level.

It must be underlined that some publications were protocols. This practice is very advantageous for research, as it provides detailed information regarding the design of the intervention and the selected electronic devices, allowing for replication and the development of new studies.

The specific uses of mobile apps through the studies were diverse. The Expecting Success study reported in a previous paper [55] that the SmartMoms remote intervention group received a Fitbit device accompanied by its app to self-monitor body weight and step counts daily. It must be pointed out that SmartMoms intervention was designed to assist an expectant mother in gaining weight within the recommended guidelines. The study by Altazan and colleagues [50] measured psychological variables such as mood, depressive symptoms, and quality of life; nevertheless, these were not treated or monitored through the use of a mobile app. The Elixir study provided its mobile group with smartphones with the preinstalled Oiva mobile app. Ahinen and colleagues [56] had previously described Oiva; based on acceptance and commitment therapy, it is a stand-alone mobile intervention for self-administered active learning skills to prevent stress and improve mental wellness. The study by Järvelä-Reijonen and colleagues [49] focused on eating behavior and diet, although neither the Oiva app nor the interventions were originally designed for these outcomes. The study by Levinson and colleagues [48] used a mobile app for recording answers to questionnaires addressing cognitions, emotions, anxiety symptoms, and eating disorder behaviors, not for delivering an intervention. It must be noticed that the target sample was people with an eating disorder, not all of them (probably only a few) with overweight/obesity and/or depression/anxiety. The RAINBOW/ENGAGE studies [44, 45] reported on an intervention designed for the management of both weight and depression. Yet, the use of a mobile app was exclusively for recording physical activity and did not include the recording of any mental dimension. Naslund and colleagues [46, 47] worked with a group of patients with severe mental illnesses from an urban community mental health center enrolled in a group behavioral weight loss program targeting fitness and healthy eating. The mobile app was used exclusively to record physical activity.

Overall, even though overweight/obesity and depression/anxiety are highly extended, increasing and related public health conditions, only a few studies provide scientific foundations relating to their simultaneous follow-up through the use of mobile apps. Results suggest the use of MyFitness, Fitbit, and Nike Fuel Ban apps to record physical activity; yet, they would need to be complemented with an app to monitor psychological wellbeing. Cognitive behavioral therapy seems the most suitable framework for interventions, aiming at long term behavioral change. Yet, as the diversity of the studies suggests, an intervention program needs to be adapted considering if its purpose is the prevention (e.g., the SmartMoms Behavioral program [50]) or the management of comorbid unhealthy weight and mental conditions (e.g., the I-CARE program [45]).

## 5. Conclusions

The prevalence and costs related to overweight/obesity and depression/anxiety are significant and likely to increase. Very often these conditions overlap; thus, it would be recommendable to treat their comorbidity simultaneously. Nevertheless, no mobile app has been designed for this purpose, which would help to reduce service provision costs and make treatment more easily accessible for patients. This opens an opportunity for mHealth research, particularly in those countries (e.g., Mexico) where these comorbid conditions prevail.

## Figures and Tables

**Table 1:** Identification features of publications.

Authors	Year of publication	Location	Type of manuscript	Name of the project	Design	Number of groups
Altazan et al.	2019	Louisiana, USA	Original article	Expecting success	Randomized controlled trial/pilot	3

Järvelä-Reijonen et al.	2018	Jyväskylä, Kuopio, and Helsinki, Finland	Original article	Elixir	Randomized controlled trial	3

Levinson et al.	2018	Missouri, USA	Original article	—	Descriptive	1

Ma et al.	2015	California, USA	Study framework and protocol	RAINBOW	Randomized controlled trial/protocol	2

Naslund, Aschbrenner, and Barre et al.	2015	New Hampshire, USA	Brief communication	—	Pre-post intervention/pilot	1

Naslund, Aschbrenner, and Scherer et al.	2016	New Hampshire, USA	Original article	—	Exploratory	1

Williams et al.	2018	California, USA	Study framework and protocol	RAINBOW/ENGAGE	Randomized controlled trial/protocol	2

*Notes*. References are presented alphabetically by the name of authors. USA: United States of America.

**Table 2:** Relevant content of publications.

*Altazan et al.*
Objective(s)
(i) To quantify changes in mental and physical quality of life and depressive symptoms across pregnancy and the postpartum period
(ii) To determine if gestational weight gain was associated with changes in mood and quality of life
(iii) To assess the effect of a behavioral intervention targeting excessive gestational weight gain on mood and quality of life
Inclusion/exclusion criteria
(i) Pregnant women
(ii) Age, 18–40 years
(iii) English speaking
(iv) With overweight or obesity
(v) Carrying viable singletons
(vi) No history or current psychotic disorder or major depressive episodes
Study sample
(i) N: 54 (initial), 43 (final)
(ii) Mean age = 29.2 years
(iii) Gestational age = 10.0 weeks
(iv) Mean weight = 83.3 kg
Intervention
Duration of approximately 18 weeks. SmartMoms®. Behavioral weight management counselling by interventionists either in a clinic-based setting (in-person) or remotely through a smartphone application (phone)
Mobile app features
Not described
Outcome measures
(i) Gestational weight gain
(ii) Quality of life/rand 12-item short form (SF-12)
(iii) Mood and depressive symptoms/Beck depression inventory II (BDI-II)
Main results
(i) No significant intervention effects
(ii) Women in the SmartMoms® intervention had less overall gestational weight gain as compared with the women in the usual care group
(iii) Higher gestational weight gain was associated with worsened mood and lower physical quality of life across pregnancy
(iv) Maternal depressive symptoms and physical health worsened significantly over time from early to late pregnancy in both the SmartMoms® and usual care group, but re-established at postpastum

*Järvelä-reijonen et al.*
Objective(s)
(i) To investigate the effects of acceptance and commitment therapy (ACT) intervention delivered in two different ways, i.e., via face-to-face group sessions and via mobile app, on reported eating behavior and diet quality among adults with psychological distress and overweight or obesity
Inclusion/exclusion criteria
(i) Age, 25–60 years
(ii) Psychologically distressed (≥3/12 points from the general health questionnaire, GHQ-12)
(iii) BMI, 27–34.9 kg/m^2^
(iv) With computer and internet access
(v) No diagnosis of severe chronic illness including eating disorder
(vi) No pregnancy or breastfeeding within the past 6 months
(vii) Disabilities/illnesses affecting substantially physiological or mental health
(viii) No participation in other intervention studies during the ongoing study
(ix) No psychotherapy or other psychological or mental treatment at least twice a month
Study sample
(i) N: 219
(ii) 85% female
(iii) Mean BMI = 31.3 kg/m^2^
(iv) Mean age = 49.5 years
Intervention
Intensive 8-week intervention participants were assigned to one of the three parallel groups: (1) ACT-based face-to-face (six group sessions led by a psychologist), (2) ACT-based mobile (one group session and mobile app), and (3) control (no intervention). The face-to-face and mobile interventions provided the same ACT program, and only the delivery method of the intervention differed. Intervention did not include nutrition education. The mobile group received smartphones with the preinstalled oiva mobile app containing 46 exercises in text and audio formats and introduction videos about the ACT skills.
Mobile app features
The oiva mobile app contains 46 exercises in text and audio formats and introduction videos about the ACT skills
Outcome measures
(i) Eating behavior
(ii) Index of diet quality (IDQ)
(iii) Intuitive eating scales (IES-1)
(iv) Alcohol use disorders identification test consumption (AUDIT-C)
(v) Three-factor eating questionnaire (TFEQ-R18)
(vi) Perceived stress scale stress (PSS)
(vii) Health and taste attitude scales, (HTAS)
(viii) 48-h dietary recall
(ix) ecSatter inventory 2.0 (ecSI 2.0)
(x) Regulation of eating behavior scale (REBS)
Main results
(i) ACT-based interventions, delivered in group sessions or by mobile app, showed beneficial effects on reported eating behavior
(ii) Beneficial effects on eating behavior were, however, not accompanied by parallel changes in diet
(iii) No statistically significant effects were found for dietary measures

*Levinson et al.*
Objective(s)
(i) To test if cognitions which occur during, or briefly after, mealtime predict subsequent eating disorder behaviors
Inclusion/exclusion criteria
(i) Clinical diagnosis of eating disorder
(ii) Recruited from an eating disorder clinic after discharge either from a residential or partial hospitalization treatment program
Study sample
(i) N: 66
(ii) Mean age, 24.9 years
(iii) 97.0% female
(iv) 86.2% European American
(v) Median BMI = 20.66
(vi) 74.2% in treatment for an eating disorder (68.2% outpatient)
(vii) 60.6% with anorexia nervosa, 21.2% with atypical anorexia nervosa, and 1.5% with bulimia nervosa
(viii) Other mental health diagnoses: 62.1% anxiety disorder, 57.6% depressive disorder, 19.7% obsessive compulsive disorder, and 10.6% posttraumatic stress disorder
Intervention
Not applicable. All procedures of this descriptive study were completed either online or through a mobile application
Mobile app features
The application consisted in a questionnaire that assesses behaviors, cognitions, and emotions before, during, and after mealtimes. It notified participants four times a day for one week and asked questions about mealtime cognitions, as well as eating disorder behaviors and anxiety
Outcome measures
(i) Eating disorder diagnostic scale (EDDS)
(ii) Eating disorder inventory-2 (EDI-2)
(iii) Daily life daily habits questionnaire
Main results
Cognitions predict subsequent eating disorder behaviors and vice versa (e.g., having high standards during a meal predicted subsequent food intake restriction, worrying about weight gain during a meal and concerns about making mistakes during the meal predicted subsequent weighing oneself, feeling fat during the meal, and preoccupation with thinness predicted subsequent excessive exercise)

*Ma et al.*
Objective(s)
(i) To evaluate the clinical and cost effectiveness and implementation potential in primary care of I-CARE (integrated coaching for better mood and weight), an integrated, technology-enhanced, collaborative care model for treating comorbid obesity and depression
Inclusion/exclusion criteria
(i) Age, 18 years and older
(ii) BMI ≥30.0 kg/m2 for non-Asians, BMI ≥27.0 kg/m2 for Asians
(iii) Clinically significant depression (≥10 points from the patient health questionnaire-9, PHQ-9)
(iv) Patient of the Palo Alto Medical Foundation ≥1 year and seen in primary care at least once in the preceding 24 months
(v) No severe mental condition other than minor or major depressive disorder and/or dysthymia, with the exception of any comorbid anxiety disorder (active suicidal ideation, bulimia nervosa, and alcohol/substance use disorder)
(vi) Not ongoing psychiatric care with a provider outside of Palo Alto Medical Foundation
(vii) No bariatric surgery within the past 12 months
(viii) No pre-existing diabetes or cardiovascular disease, diagnosis of cancer, and/or severe medical comorbidities that require aggressive treatment
(ix) No diagnosis of a terminal illness and/or residence in a long-term care facility
(x) No cognitive impairment based on the callahan 6-item screener
(xi) Capable of speaking, reading, and understanding English
(xii) With reliable telephone service and/or regular internet access
(xiii) No plan to move out of the area or transfer care outside the Palo Alto Medical Foundation
(xiv) Not currently pregnant, lactating, or planning to become pregnant
(xv) Not enrolled, or planning to enroll, in another research study
(xvi) No family/household member of another participant or of a staff member
Study sample
N: target sample size of 404 participants
The gender and minority racial/ethnic composition of the target enrollment population is estimated to be 64% female, 10% non-Hispanic black, 18% Hispanic/Latino, and 44% Asian
Intervention
Duration of 12 months. Participants will be randomized to usual care enhanced with the provision of a pedometer and information about the health system's services for mood or weight management (control) or with the I-CARE program (intervention). The I-CARE program integrates the diabetes prevention program-based group lifestyle balance™ (GLB) program for weight loss and cardiometabolic risk reduction with the PEARLS program for collaborative stepped depression care, which uses problem solving therapy combined with behavioral activation, intensified with stepwise increases in doses and number of antidepressant medications as needed
Mobile app features
Participants will wear a fitbit pedometer and log their weight on the fitbit website or mobile application; also, they will log their minutes of physical activity and dietary intake using MyFitnessPal website or app
Outcome measures
(i) BMI
(ii) Quality of life (short Form-8 health survey, euro-qol-5d-5 L)
(iii) Depression (depression symptom Checklist-20)
(iv) Impact of obesity on psychosocial functioning (obesity-related problem scale)
(v) Anxiety (generalized anxiety disorder scale (GAD-7), panic
(vi) Functional disability (Sheehan disability scale)
(vii) Disorder module of the mini-international neuropsychiatric interview)
(viii) Direct medical costs and direct nonmedical costs
Main results
Not applicable

*Naslund, Aschbrenner, Barre et al.*
Objective(s)
(i) To assess the feasibility of using popular mHealth technologies for activity tracking among overweight and obese individuals with severe mental illness
Inclusion/exclusion criteria
(i) Age, 21 years and older
(ii) BMI ≥ 25 kg/m^2^
(iii) Impairment in multiple areas of functioning
(iv) Diagnosis of severe mental illness (schizophrenia spectrum disorder, bipolar disorder, or major depressive disorder)
Study sample
(i) N: 10 (initial), 9 (final)
(ii) Mean age = 47.7
(iii) Mean BMI = 42.4 kg/m^2^
(iv) 90% female
(v) 90% white
(vi) Diagnosis: 3 with schizophrenia, 1 with bipolar disorder, 6 with major depressive disorder
Intervention
The weight loss program was adapted from the in SHAPE intervention for people with severe mental illness and consisted of weekly peer-led group and individual exercise and nutrition education sessions, as well as individual meetings with a certified fitness trainer. The intervention lasted between 80 and 133 days
Activity tracking devices, smartphones, and mobile apps were provided
Mobile app features
Participants were provided with iPhone 4S smartphones and one of two commercially available devices: FitBit zip or nike inc. FuelBand. Both are small size accelerometers that track steps, distance, and calories burned. Each device syncs wirelessly with its own smartphone application that reward milestones, track activity over time, and allow users to compare steps and progress with others through a closed social network
Outcome measures
(i) Feasibility (frequency of device use)
(ii) Acceptability (follow-up semistructured interviews regarding behavior, experiences, and preferences)
Main results
Participants reported high satisfaction, stating the devices were easy to use, helpful for setting goals, motivational, and useful for self-monitoring. Several participants liked the social connectivity feature of the devices where they could see each other's progress on the smartphone application, noting that “friendly” competition increased motivation to be more physically active

*Naslund, Aschbrenner, Scherer et al.*
Objective(s)
(i) To examine whether daily step count measured using fitbit wearable devices was associated with weight loss and improved fitness among individuals with serious mental illness enrolled in a 6-month lifestyle program
Inclusion/exclusion criteria
(i) Age, 21 years and older
(ii) BMI ≥ 30 kg/m^2^
(iii) English speaker
(iv) Diagnosis of severe mental illness (schizophrenia, schizoaffective disorder, major depressive disorder, or bipolar disorder)
(v) Stable pharmacological treatment status defined (i.e., receiving the same psychiatric medications over the prior 2 months)
(vi) No medical contraindication to weight loss
(vii) Not pregnant or planning to become pregnant within the next 6 months
(viii) No current diagnosis of an active alcohol-use or substance-use disorder
Study sample
(i) N: 43 (initial), 34 (final)
(ii) Mean age = 50.2
(iii) Mean BMI = 38.5 kg/m^2^
(iv) 61.8% female
(v) 100% non-Hispanic white
(vi) Diagnosis: 8 with schizophrenia, 17 with major depressive disorder, and 9 with bipolar disorder
Intervention
Duration of 6 months. The group-based lifestyle behavioral program focused on achieving weight loss through healthy eating and increasing physical activity through weekly sessions led by lifestyle coaches
Mobile app features
The fitbit zip is a compact wearable accelerometer that tracks number of steps, and it synchs wirelessly with a free companion smartphone application. The fitbit rewards milestones such as reaching daily step goals with colourful trophies, and it allows users to compare steps and progress with other through the smarthphone application
Outcome measures
(i) Daily step count
(ii) Weight
(iii) Fitness (6-minute walk test)
(iv) Change in fitness (calculated as the change in feet on the 6-minute walk test from baseline to 6-months)
Main results
At 6 months, higher average daily step count was associated with greater weight loss but not improved fitness

*Williams et al.*
Objective(s)
(i) To identify assays that engage emotion, cognition, and self-reflection aspects of self-regulation within lab-based, VR, and naturalistic settings, to understand the relations between assays taken at different levels of measurement, and to evaluate the extent to which these assayed self-regulation targets predict and/or mediate adherence to the intervention and mood and weight outcome
Inclusion/exclusion criteria
(i) Adult patients receiving primary care at Palo Alto Medical Foundation
(ii) Weight <350 pounds
(iii) With comorbid depression and obesity
(iv) With no significant medical or psychiatric comorbidities
(v) No MRI contraindications
vi) No traumatic brain injuries
(vii) No tumor or any other known structural abnormality in brain
Study sample
N: minimum target sample size of 100 participants
Intervention
The ENGAGE (engaging self-regulation targets to understand the mechanisms of behavior change and improve mood and weight outcomes) study is embedded within and operationally linked to the RAINBOW (research aimed at improving both mood and weight) randomized controlled trial. See the description of the RAINBOW study by Ma and colleagues mentioned above
Mobile app features
A mobile application will be used exclusively for the naturalistic assays. The mindstrong data collection application will be installed on a participant's smart phone and will provide continuous passive naturalistic sampling of 288 phone-use feature variables throughout the two-year study period. Mindstrong's technology and algorithms have shown that a user's day-to-day smartphone keyboard and swipe-tap interactions contained repeated psychometric challenges capable of approximating gold-standard neuropsychological tests across a battery of domains
Outcome measures
Assays of three self-regulation targets (emotion, cognition, and self-reflection) will be collected in multiple settings: neuroimaging and behavioral lab-based measures, virtual reality, and passive smartphone sampling. Through data analysis, researchers will identify which targets relate most to behavioral and health outcomes, which change together, and which predict who will achieve behavioral change following an intervention and why
The mindstrong app used for the passive smartphone sampling targeting regulation of emotion, cognition, and self-reflection will record word frequencies collected from input text in emails, text messages, or search terms as well as punctuation usage indicative of emotional states, and behavioral changes will be inferred from changes in social activity, such as incoming and outgoing calls and messages
Main results
Not applicable

## Supplementary Materials

Supplementary Materials Appendix 1. Number of results according to introduced keywords and database.
