# Supplementary material for: Mobile Apps for the Management of Comorbid Overweight/Obesity and Depression/Anxiety: A Systematic Review
Source: J Healthc Eng. 2020 Jan 23;2020:9317179. doi: 10.1155/2020/9317179 (PMC7003257; doi:10.1155/2020/9317179)
Supplement: Supplementary Materials — Appendix 1. Number of results according to introduced keywords and database. [file 9317179.f1.docx]

| Appendix 1. Number of results according to introduced keywords and database | | | | | | | |
| --- | --- | --- | --- | --- | --- | --- | --- |
| Introduced keywords | | | Database | | | | |
| First | Second | Third | Pubmed | PsycNet | Web of Science | ResearchGate | Lilacs |
| Obesity | Anxiety | Mobile app (application) | 3 | 52 | 2 | 7 | 1 |
|  |  | Smartphone app (application) | 1 | 74 | 0 | 0 | 1 |
|  |  | Android app (applicattion) | 0 | 2 | 0 | 0 | 0 |
|  |  | iOS app (application) | 0 | 1 | 0 | 0 | 0 |
|  |  | Mobile health app (application) | 1 | 3 | 2 | 0 | 0 |
|  |  | mHealth app (application) | 1 | 12 | 0 | 6 | 0 |
| Obesity | Depression | Mobile app (application) | 11 | 61 | 14 | 15 | 7 |
|  |  | Smartphone app (application) | 3 | 96 | 10 | 0 | 5 |
|  |  | Android app (applicattion) | 1 | 2 | 1 | 0 | 1 |
|  |  | iOS app (application) | 0 | 1 | 0 | 1 | 0 |
|  |  | Mobile health app (application) | 5 | 7 | 10 | 15 | 5 |
|  |  | mHealth app (application) | 4 | 14 | 5 | 4 | 5 |
| Overweight | Anxiety | Mobile app (application) | 2 | 25 | 1 | 2 | 0 |
|  |  | Smartphone app (application) | 1 | 56 | 1 | 0 | 1 |
|  |  | Android app (applicattion) | 0 | 1 | 0 | 0 | 0 |
|  |  | iOS app (application) | 0 | 0 | 0 | 0 | 0 |
|  |  | Mobile health app (application) | 1 | 1 | 1 | 2 | 0 |
|  |  | mHealth app (application) | 1 | 6 | 0 | 0 | 1 |
| Overweight | Depression | Mobile app (application) | 8 | 36 | 7 | 6 | 3 |
|  |  | Smartphone app (application) | 1 | 54 | 5 | 0 | 2 |
|  |  | Android app (applicattion) | 0 | 1 | 0 | 0 | 0 |
|  |  | iOS app (application) | 0 | 0 | 0 | 0 | 0 |
|  |  | Mobile health app (application) | 2 | 1 | 5 | 5 | 2 |
|  |  | mHealth app (application) | 2 | 5 | 2 | 1 | 2 |
| Total by database | | | 48 | 511 | 66 | 64 | 36 |
| Duplicated results by database | | | 37 | 379 | 33 | 46 | 26 |
| Final total by database | | | 11 | 132 | 33 | 18 | 10 |
